# The Impact of Acquired Hypothyroidism on the Growth and Metabolic Profiles of Pediatric Patients: A Retrospective Monocentric Study

**DOI:** 10.3390/children12030272

**Published:** 2025-02-24

**Authors:** Elena Malavolta, Ignazio Cammisa, Giulia Rotunno, Lucia Celeste Pane, Federica Arzilli, Giorgio Sodero, Donato Rigante, Clelia Cipolla

**Affiliations:** 1 Department of Woman and Child Health and Public Health, Fondazione Policlinico Universitario A. Gemelli IRCCS, 00168 Rome, Italyignazio.cammisa01@icatt.it (I.C.); giulia.rotunno01@icatt.it (G.R.); luciaceleste.pane01@icatt.it (L.C.P.); donato.rigante@unicatt.it (D.R.);; 2 Pediatric Department, Perrino Hospital, 72100 Brindisi, Italy; 3 Pediatric Endocrinology Unit, Perrino Hospital, 72100, Brindisi, Italy; 4 Department of Life Sciences and Public Health, Università Cattolica del Sacro Cuore di Roma, 00168 Rome, Italy

**Keywords:** hypothyroidism, levothyroxine, growth, pediatric endocrinology

## Abstract

***Background*:** Hypothyroidism is the most common thyroid dysfunction in childhood, resulting from the decreased biological activity of thyroid hormones in tissues. Pediatric patients with hypothyroidism, when left untreated or when thyroid hormone levels fail to normalize despite treatment, may exhibit various complications such as growth retardation, obesity, and hypercholesterolemia. ***Aim*:** We conducted a monocentric retrospective study to evaluate potential differences in obesity rates and auxological parameters between healthy patients and children with hypothyroidism undergoing levothyroxine replacement therapy. Additionally, we examined possible differences in lipid and glucose metabolism between the two groups. ***Materials and Methods*:** We collected and analyzed data from the electronic medical records of 108 patients who were regularly followed up for thyroid dysfunction at the Pediatric Endocrinology Unit of the *Fondazione Policlinico Universitario A. Gemelli IRCCS* from January 2016 to June 2024. We also included 104 healthy controls who underwent thyroid function testing during the same period, followed up in the same department for regular auxological check-ups. ***Results*:** Our findings revealed that patients with acquired hypothyroidism had a lower height z-score compared to healthy controls (t(210) = −2.6; *p* = 0.01). Additionally, they exhibited higher blood glucose and triglyceride levels, although these values remained within the normal range. ***Conclusions*:** We highlight the critical importance of the early diagnosis of hypothyroidism to initiate levothyroxine replacement therapy promptly and mitigate the long-term effects of hypothyroidism on children’s growth.

## 1. Introduction

Thyroid hormones have important physiologic functions in nearly every organ system of the human body; the critical role of thyroid hormones in growth and in physical and neurologic development lends particular importance to the prompt diagnosis and proper treatment of hypothyroidism in infants and children [1]. The most common symptoms of hypothyroidism in children and adolescents include growth retardation, delayed psychomotor and cognitive development, excessive fatigue, weight gain, dry skin and brittle hair, bradycardia, cold intolerance, and constipation.

The thyroid gland is composed of two lobes which are connected by the central isthmus located in the lower portion of the neck. The gland develops from an endodermal diverticular outgrowth of the primitive pharynx, and the thyroid diverticulum first forms at the end of the fourth week of development as a solid, proliferating mass of endoderm that migrates down through the developing neck via the thyroglossal duct toward its eventual home. The only remaining aspect of the thyroid’s embryonal development is the foramen cecum at the base of the developed tongue. The isolated thyroid gland develops two distinct lobes connected by an isthmus of tissue by this time and continues to descend and reach its final destination by the end of the seventh week of development. The primary secretory product of the thyroid gland is inactive thyroxine, or T4, a prohormone of triiodothyronine, or T3. T4 is converted to T3 peripherally by type 1 deiodinase in tissues with high blood flow, such as the liver and kidneys [2].

These iodothyronines are composed of thyroglobulin and iodine. Thyroglobulin is formed from amino acids in a basal to apical fashion within the thyroid cells. Thyroglobulin is then secreted into the follicular lumen and is enzymatically combined with iodine to form iodinated thyroglobulin. Endosomes containing this iodinated thyroglobulin then fuse with lysosomes, which enzymatically release the thyroglobulin from the resultant thyroid hormone [2].

Primary hypothyroidism is the most common thyroid dysfunction in childhood, resulting from the decreased biological activity of thyroid hormones in tissues, and could be caused by an organic or functional alteration in the thyroid gland, with an insufficient production and/or secretion of thyroid hormones: thyroxine [fT4] and triiodothyronine [fT3]. Less common causes of hypothyroidism may include conditions such as resistance to thyroid hormone action (peripheral hypothyroidism) or the accelerated degradation of thyroid hormones in peripheral tissues (consumption hypothyroidism) [3].

Hypothyroidism can be classified as either congenital or acquired. Congenital hypothyroidism (CH) arises from defective thyroid hormone production present from birth. Furthermore, there are three major types of hypothyroidism due to the location of the defect. Hypothyroidism due to abnormalities in the thyroid gland itself is primary hypothyroidism. Secondary hypothyroidism is caused by a deficiency in thyroid stimulating hormone (TSH) released from the anterior pituitary, and tertiary hypothyroidism is caused by a deficiency in thyrotropin-releasing hormone (TRH) released from the hypothalamus, which reduces the amount of TSH released. Both secondary and tertiary hypothyroidism are central hypothyroidism.

Most congenital hypothyroidism is sporadic and usually occurs due to thyroid gland dysgenesis. In some cases, hypothyroidism is familial, resulting from inborn errors leading to the altered synthesis of thyroid hormones. Familial hypothyroidism can be due to a number of genes and can have either an autosomal recessive or autosomal dominant pattern. The mutation of various genes can lead to hereditary congenital hypothyroidism, for example, paired box gene 8 (*PAX8*) and thyroid stimulating hormone receptor (*TSHR*), which are important in thyroid gland development; dual oxidase 2 (*DUOX2*), solute carrier family 5 member 5 (*SLC5A5*), thyroglobulin (*TG*), and thyroid peroxidase (*TPO*), which are involved in the synthesis of thyroid hormones; and thyroid stimulating hormone beta (*TSHB*), which has a role in the stimulation of hormone production, and mutations in this gene lead to central hypothyroidism [4].

Most infants with CH are identified by the newborn screening in the first weeks after birth, so early thyroxine replacement therapy can prevent intellectual disability in a child [5].

In general, acquired hypothyroidism is not associated with intellectual disability, except in early-onset cases appearing in the first two to three years of life. Globally, iodine deficiency is the primary cause of hypothyroidism in regions with insufficient iodine intake. However, in industrialized countries, where iodine is abundant in the diet, autoimmune disorders are the predominant causes [4]. Hashimoto’s thyroiditis, or chronic lymphocytic thyroiditis [CLT], is the most common autoimmune disorder and the leading cause of acquired hypothyroidism in both adults and children or adolescents [6,7]. Acquired hypothyroidism frequently presents at between 9 and 11 years of age and is rarely seen before 4 years of age. Approximately 80% of children and adolescents are asymptomatic at the time of diagnosis. Children with moderate-to-severe hypothyroidism, in both congenital and acquired forms, often require medical attention to assess poor growth, constipation, lethargy, and/or dry skin [8,9]. The distinctive characteristic of hypothyroidism during childhood and adolescence, especially if acquired and with a belated diagnosis that does not allow the adequate and rapid treatment of the thyroid hormone deficiency, is its profound impact on skeletal growth, maturation, and pubertal development, with potential repercussions on the final adult height [10,11,12]. Hypothyroidism can lead to weight gain; therefore, it may have an important role in the development of obesity. Childhood obesity may be the most serious health issue in developing countries [13].

Since thyroid hormones regulate the basal metabolic rate and thermogenesis, lipid and glucose metabolism, and food intake and fat oxidation, involving metabolic pathways relevant to resting energy expenditure (REE), it is not surprising that patients with thyroid disease may show changes in their body weight and body composition, thermogenesis, and REE apart from physical activity [14]. Hypothyroidism is usually associated with weight gain or decreased thermogenesis and has been shown to correlate with a higher body mass index [BMI] and higher prevalence of obesity [15]. Moreover, hypothyroidism is a leading cause of hypercholesterolemia, characterized by elevated levels of low-density lipoprotein cholesterol [LDL-C] and caused by a decreased catabolism of LDL because of a reduction in the number of LDL receptors on liver cell surfaces mediated by T3, and hypertriglyceridemia, caused by a reduced removal rate of triglycerides from plasma due to the decreased activity of hepatic triglyceride lipase [16]. Obese children are at a high risk of developing additional disorders, such as diabetes mellitus, dyslipidemia, hypertension, and cardiovascular disease, or being diagnosed as having metabolic syndrome. In addition, it has been estimated that children who are obese during childhood are more likely to become obese adults [13,14,15,16,17].

Furthermore, obesity itself is associated with subclinical hypothyroidism. Indeed, slight hyperthyrotropinemia and a moderate increase in free T3 (fT3) are the most common hormonal abnormalities found in obese children: they correlate positively with BMI and the degree of obesity [18,19], while no difference has been found for fT4. Higher TSH levels were explained by the role of leptin which is predominantly released by adipocytes and stimulates the secretion of thyrotropin-releasing hormone [TRH] and TSH by the hypothalamic–pituitary axis [20,21]. At the same time, leptin influences the responsiveness of the thyroid gland to TSH and the activity of deiodinases, as shown by the increase in total T3 and free T3 [fT3]. The high conversion rate of T4 to T3 is interpreted as a defense mechanism, to counteract the accumulation of fat by increasing the energy expenditure, basal metabolic rate, and total energy expenditure [17,22,23]. Consistent with this interpretation, some studies have reported that weight loss causes a significant decrease in serum fT3 and TSH [24,25,26,27,28].

To address current gaps in the literature, we conducted a monocentric retrospective study to evaluate potential differences in obesity rates and auxological parameters between healthy patients and children with hypothyroidism undergoing levothyroxine replacement therapy. We aimed to investigate whether children with hypothyroidism undergoing adequate levothyroxine replacement therapy can achieve a risk of obesity and auxological alterations comparable to those of the general population.

## 2. Materials and Methods

### 2.1. Study Characteristics

We conducted a single-center observational retrospective study investigating the metabolic and anthropometric differences between patients with acquired hypothyroidism and healthy controls; all parents of the recruited patients were informed about the purpose of this study and signed an informed consent form to authorize access to their children’s medical records and allow their personal data to be processed. None of the parents refused to participate in our study. According to the General Authorization to Process Personal Data for Scientific Research Purposes [Authorization No. 9/2014], ethics committee approval is not required for retrospective studies that use identification codes preventing the tracing of patients’ data.

### 2.2. Patients’ Selection

We collected and analyzed auxological and metabolic data from the electronic medical records of patients regularly followed up for thyroid dysfunction at the Pediatric Endocrinology Unit of the Fondazione Policlinico Universitario A. Gemelli IRCCS from January 2016 to June 2024. We also included age-matched healthy controls who underwent thyroid function testing during the same period, followed up in the same department for regular auxological check-ups. We also analyzed the same parameters in a control group consisting of healthy patients with no endocrine disorders, who were regularly monitored at the pediatric endocrinology clinic for routine auxological evaluations.

### 2.3. Inclusion Criteria for Study Group

The inclusion criteria were as follows:

- Age under 20 years;
- Coded diagnosis of acquired hypothyroidism undergoing levothyroxine replacement therapy;
- Patients who at diagnosis had altered TSH and FT4 values, defined by the following cut-off points: TSH > 3.50 µUI/mL and fT4 < 8.5 pg/mL;
- Good adherence to levothyroxine therapy (as reported by the patient and/or parents and confirmed through analysis of the thyroid hormone profile) and duration >6 months;
- Absence of other medical conditions and/or pharmacological treatments that could potentially affect the hypothalamic–pituitary axis.

### 2.4. Exclusion Criteria for Study Group

The exclusion criteria were as follows:

- Presence of genetic disorders or being small for gestational age;
- Patients born small for gestational age;
- Patients affected by congenital hypothyroidism;
- Patients affected by cancer.

### 2.5. Data Extraction and Interpretation

Data about laboratory and anthropometric parameters listed in [Table 1] were collected in an electronic database for the subsequent statistical analysis.

Height was measured by a single endocrinologist using a stadiometer, with the subject having bare feet, looking straight ahead, and standing with their back against the wall. Weight was measured using an electronic scale, and BMI was calculated as weight in kilograms divided by the square of height in meters. Z-score and percentiles for anthropometric data were calculated according to Centers for Disease Control [CDC] growth charts. The target height was calculated with the Tanner method. Patients with a BMI percentile ≥ 95th [or a value ≥ 30] for sex and age were defined as obese, whereas patients with a BMI between the 85th and 95th percentiles [or a value between 25 and 29.9] were defined as overweight.

Blood exams for hormonal and biochemical parameters were routinely analyzed by the clinical service laboratory of the Policlinico Universitario Agostino Gemelli Hospital; more specifically, patients underwent follow-up blood tests every 3–6 months, always in the morning and fasting. Thyroid hormone levels [TSH, fT3, fT4] and biochemical markers describing metabolic health, including blood glucose [mg/dL], total cholesterol [mg/dL], high-density lipoprotein cholesterol [HDL-C, mg/dL], low-density lipoprotein cholesterol [LDL-C, mg/dL], and triglyceride concentrations [mg/dL], were selected for analyses. The TSH, fT3, and fT4 levels were measured by the chemiluminescence immunoassay (CLIA). The reference values, which are considered normal according to the local laboratory, were as follows: TSH: 0.35–3.50 µUI/mL; fT3: 3.3–4.8 pg/mL; and fT4: 8.5–14.3 pg/mL. Patients were considered euthyroid if their serum fT4 and TSH levels were in the normal range. We also collected antibodies against thyroid peroxidase [TPOab] and human thyroglobulin [hTGab] measured by the chemiluminescence method when available. A TPO-ab level > 60 IU/mL and an hTGab level > 4.5 IU/mL were considered positive.

According to the Italian Society of Diabetology [24], we considered fasting glucose values < 100 mg/dL as normal. According to the Expert Panel on Integrated Guidelines for Cardiovascular Health and Risk Reduction in Children and Adolescents [25], we considered plasma lipids as follows: total cholesterol: high if >200 mg/dL; LDL cholesterol: high if >130 mg/dL; HDL cholesterol: low if <40 mg/dL; and triglycerides: high if ≥130 mg/dL.

### 2.6. Statistical Analysis

All the analyzed data were collected in an Excel database, which was subsequently used for statistical analysis. Categorical variables were expressed as numbers; continuous variables were expressed as the mean and standard deviation. T-test and Chi-square test were performed to determine significant differences in anthropometric and laboratory variables between patients with and without thyroid dysfunction. Statistical analysis was performed using IBM SPSS Statistics software version 25.0 [IBM Corporation, Armonk, NY, USA]. Statistical significance was determined as alpha <0.05 in all cases.

## 3. Results

We collected and analyzed data from the electronic medical records of 108 patients who were regularly followed up for thyroid dysfunction at the Pediatric Endocrinology Unit of the Fondazione Policlinico Universitario A. Gemelli IRCCS from January 2016 to June 2024. We also included 104 healthy controls who underwent thyroid function testing during the same period, followed up in the same department for regular auxological check-ups.

We included pediatric patients [defined as individuals aged 0 to 18 years old] and two patients who, at the time of this study, exceeded the pediatric age range. However, since this is a longitudinal study and these two patients were diagnosed at the ages of 13 and 12 years old, the data collected from their most recent follow-up were included.

At the time of diagnosis, all patients were in the prepubertal stage. All the recruited patients with hypothyroidism were on thyroid hormone replacement therapy; none of the subjects analyzed were taking any other medications that could influence the hypothalamic–pituitary axis.

Among the participants, 149 [70.2%] were females, and 63 [29.7%] were males, with a mean age of 9.7 years old [ranging from 1 to 21]. Data about anthropometric parameters [age, height, weight, BMI, and respective z-scores] and thyroid function [TSH, fT3, fT4] were collected for all patients. Healthy controls had an overall normal TSH for their age [26]. Additionally, metabolic data were available for some patients, including glucose [125 patients], triglycerides [104 patients], total cholesterol [109 patients], HDL cholesterol [96 patients], and LDL cholesterol [97 patients].

According to our classification criteria, only four patients [1.8%] had pathological glucose levels [two patients with thyroid dysfunction and two healthy controls]. Eleven patients [5.1%] had pathological total cholesterol levels [seven patients with thyroid dysfunction and four healthy controls]. Six patients [2.8%] had pathological LDL cholesterol levels [four patients with thyroid dysfunction and two healthy controls]. Twelve patients [5.6%] had pathological triglyceride levels [three patients with thyroid dysfunction and nine healthy controls]. Twenty-three patients [10.8%] had pathological HDL cholesterol levels [eight patients with thyroid dysfunction and fifteen healthy controls].

**Figure 1:**
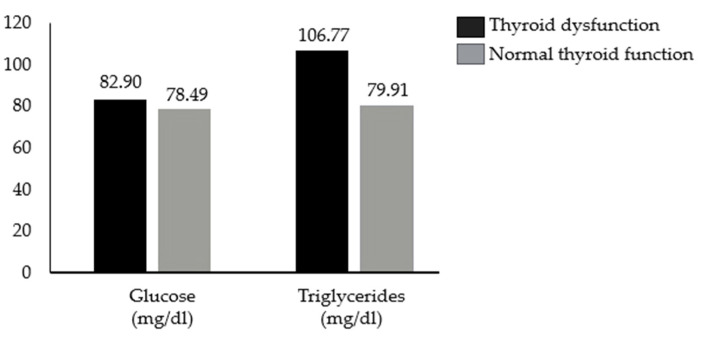
Differences in the mean glucose and triglycerides between patients with and without thyroid dysfunction in our cohort.

The Student T-test was performed to determine whether the anthropometric and laboratory variables were statistically different in patients with and without thyroid dysfunction. For laboratory parameters, no statistically significant differences were found for total cholesterol, LDL cholesterol, and HDL cholesterol. Statistically significant differences were found for glucose [t [123] = 2; *p* = 0.045] and triglycerides [t [102] = 2; *p* = 0.046], both of which had higher values in the hypothyroidism group [Figure 1].

**Figure 2:**
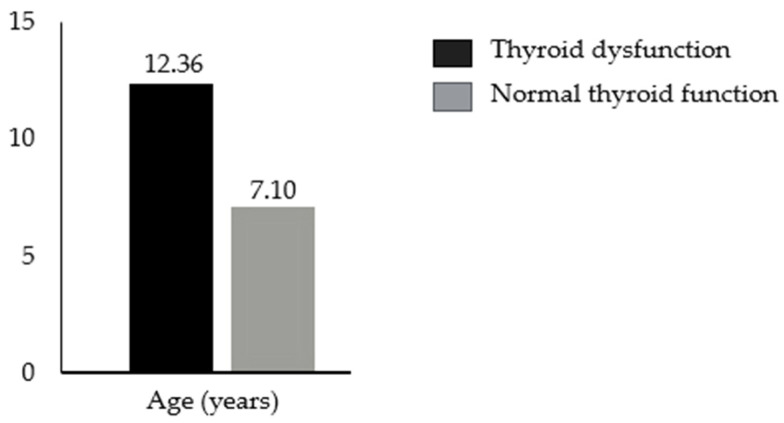
Differences in mean age between patients with and without thyroid dysfunction in our cohort.

**Figure 3:**
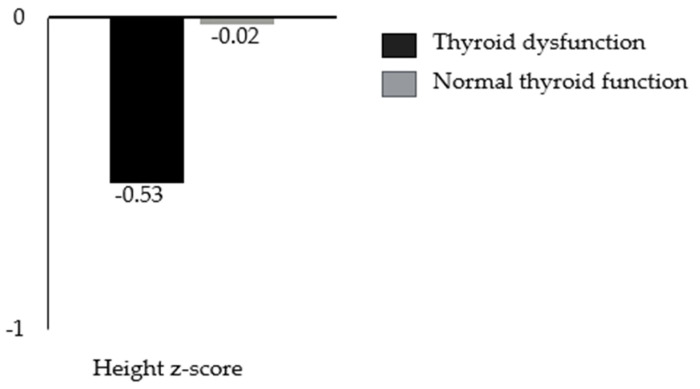
Differences in mean height z-scores between patients with and without thyroid dysfunction in our cohort.

Although the differences observed are statistically significant, we do not consider them to be clinically meaningful because most of the values were in the normal range, and for pathological values, no significant associations with one or the other group were found according to the Chi-square test. For the other parameters, statistically significant differences were found for the age [t [204] = 6.7; *p* < 0.001] and height z-scores [t [210] = −2.6; *p* = 0.01] [Figure 2 and Figure 3].

The mean age was higher in the thyroid dysfunction group [12.3 ± 5.2 years] than in the normal thyroid group [7.1 ± 6 years]. The mean height z-score was higher in the normal thyroid function group [−0.02 ± 1.39] than in the thyroid dysfunction group [−0.53 ± 1.44]. These differences in z-scores for height were not explained by differences in genetic targets. The mean genetic target was 159.98 cm for women with thyroid dysfunction and 160.1 cm for women with normal thyroid function. The mean genetic target was 172.0 cm for males with thyroid dysfunction and 172.06 cm for males with normal thyroid function. The T-test revealed no statistically significant differences in the genetic target values between boys and girls in the two groups [*p* = 0.109 for females, *p* = 0.083 for males].

No statistically significant differences were found for the BMI z-scores between the normal thyroid function group [mean 0.58] and the thyroid dysfunction group [mean 0.49] [*p* = 0.615]. No statistically significant differences were found for the weight z-scores between the normal thyroid function group [mean 0.46] and the thyroid dysfunction group [mean 0.15] [*p* = 0.135] [Table 2].

## 4. Discussion

We conducted a monocentric retrospective study to analyze any potential anthropometric and metabolic differences between pediatric patients with acquired hypothyroidism undergoing levothyroxine replacement therapy and a control group of healthy children without endocrine disorders. Our findings revealed that patients with acquired hypothyroidism had a lower height z-score compared to healthy controls. Additionally, they exhibited higher blood glucose and triglyceride levels, although these values remained within the normal range.

These alterations, particularly those concerning the height z-scores, may be linked to the role of thyroid hormones in growth and pubertal development; congenital and/or acquired hypothyroidism is, in fact, one of the leading causes of short stature [25,27,28]. It can be hypothesized that levothyroxine replacement therapy, while ensuring a thyroid profile within normal limits, may not fully replicate the thyroid gland’s natural effect on growth [28].

Hypothyroidism is a known cause of growth retardation due to the action of thyroid hormones on tissue receptors and the deiodinase enzyme system, which is another intrinsic regulator of thyroid hormone availability essential for normal growth and development [28,29]. Earlier detection of congenital hypothyroidism is necessary to prevent the associated complications such as deficits in physical development with incomplete catch-up growth and severe neurological impairments [29].

Newborn screening for congenital hypothyroidism has reduced the mean age at diagnosis and improved auxologic and neurologic outcomes [30]. Previous studies have already investigated the final height of children with congenital hypothyroidism, evaluating the importance of prognostic factors and adherence to treatment [31]. The most important clue significantly related to final height in congenital hypothyroidism is the age at the onset of treatment, as a delay in treatment may lead to the loss of growth potential of the epiphyseal cartilage [32]. Other studies have shown that not only the age at the start of treatment but also the dosage of levothyroxine may have an influence on length growth: children with congenital hypothyroidism diagnosed during newborn screening and treated early with a sufficiently high daily dose of fT4 grow normally and reach a normal adult height [33]. On the other hand, some further studies reported that the age at diagnosis and initial dose of fT4 had a poor effect on final height [30].

Our investigation revealed a lower height z-score in children with hypothyroidism undergoing replacement therapy (−0.53 ± 1.44) compared to the control group (−0.02 ± 1.39). However, no differences were observed between the groups in relation to their genetic target, for both males and females. We emphasize that all our patients with hypothyroidism consistently adhered to the prescribed therapy, with no compliance issues, and that their thyroid function tests remained in the normal range. The results of our investigation align with those found in other studies. Becker et al. [12] conducted a similar analysis, highlighting the negative effects of acquired hypothyroidism on growth, the severity of which was significantly correlated with the severity of hypothyroidism at diagnosis. Although they showed that most patients under adequate fT4 substitution experienced catch-up growth, defined as a linear growth rate higher than expected for age, the majority of patients still had a negative value at the end of follow-up, suggesting that adequate treatment with fT4 prevents short stature in the vast majority of children but cannot fully compensate, resulting in a minor height loss [12]. Therefore, the irreversibility of height loss is consistent with the findings of Pantsiotou and Rivkees, who showed reduced height growth in children with juvenile hypothyroidism several years before diagnosis and a permanent definitive height deficit in juvenile hypothyroidism [34]. Vincent et al. examined catch-up growth in a multicenter study of children referred due to growth retardation and diagnosed with Hashimoto’s hypothyroidism, reporting a median height at diagnosis of −2.7 SDS with a significant height loss of 2.5 SDS compared to height before growth retardation and inadequate catch-up growth after thyroid hormone replacement [35]. Our data could be explained by the delay in the diagnosis of acquired hypothyroidism and the deficit related to the duration of thyroxine deficiency before treatment, as described in the literature [34]. Thus, in the event of growth arrest or slowdown, it is essential to investigate thyroid function in order to anticipate the diagnosis and start replacement treatment promptly.

Our investigation also highlights the absence of differences in the z-score for weight and BMI in children with hypothyroidism under replacement therapy compared to the control group. It is well known that hypothyroidism can be associated with weight gain, decreased thermogenesis, and a decreased metabolic rate, and it has been shown to correlate with a higher BMI and a higher prevalence of obesity [15].

A bidirectional relationship between excessive weight and thyroid disease has been proposed in several studies. First, hypothyroidism may favor weight gain by reducing the basal metabolic rate; secondly, obesity and increased leptin levels play an immunomodulatory and pro-inflammatory role that triggers autoimmunity. Furthermore, the excess weight alone can elevate TSH levels even in the absence of autoimmunity markers, increasing the controversy regarding the cause–effect role of each player [36,37,38]. Jonklaas et al. showed that the health benefits from the treatment of hypothyroidism could include a more favorable lipid profile, decreased progression of coronary artery disease, improved cardiac function, normalized metabolism and weight homeostasis, normalized reproductive function, and improved memory. Compared with untreated patients, patients treated for their hypothyroidism have been shown to have decreased cardiovascular disease risk [39]. Our investigation demonstrated that hypothyroid patients maintaining a state of euthyroidism through replacement therapy can achieve weight, BMI, and cholesterol levels comparable to those of the non-hypothyroid population. This underscores the importance of close follow-up for hypothyroid patients, with repeated measurements of thyroid hormones over time to promptly identify any need for therapy adjustments.

Our investigation also showed statistically significant differences in the triglyceride and blood glucose levels of children with hypothyroidism under replacement therapy compared to the control group. These statistically significant differences are, however, clinically irrelevant. The levels of triglycerides and blood glucose among the groups are all within values considered normal, so the observed difference still falls within the normal range. Current evidence indicates an increased risk of type 2 diabetes in individuals with hypothyroidism and lower fT4 levels within the reference range [40]. The association between thyroid function and type 2 diabetes has long been hypothesized due to the effects of thyroid hormones on carbohydrate and lipid metabolism, as well as insulin secretion [41]. However, De Vries et al. demonstrated that TSH levels within the reference range are not associated with type 2 diabetes [42]. Therefore, our findings underscore the importance of establishing and continuously monitoring replacement therapy in patients with primary hypothyroidism.

Our study has some limitations: it is a single-center retrospective study, and the data are derived from Caucasian patients only. Therefore, our findings may not be generalizable to the overall pediatric population. We did not calculate the sample size for statistical power analysis; therefore, it is possible that our results may not be generalizable to the broader population; it is possible that a more extensive analysis conducted on a larger sample with a smaller age difference could yield different results. Despite these limitations, we recruited a significant number of pediatric patients with hypothyroidism, and our results lay the groundwork for future multicenter trials aimed at investigating the auxological and metabolic characteristics of children with hypothyroidism undergoing hormone replacement therapy.

## 5. Conclusions

In our retrospective analysis, we demonstrated that children with acquired hypothyroidism, despite receiving levothyroxine replacement therapy and achieving normal thyroid function, had significantly lower height z-scores compared to healthy controls. This difference can be attributed to the impaired growth rate during the pre-diagnosis phase of hypothyroidism. Although these children maintain a normal growth rate once euthyroidism is restored, they do not recover the height deficit incurred during the undiagnosed period. Furthermore, while these patients displayed elevated blood glucose and triglyceride levels, these values remained within the normal range. Our findings underscore the crucial importance of the early detection and treatment of hypothyroidism, as the timely initiation of levothyroxine therapy can help prevent the long-term negative effects of hypothyroidism on growth and metabolic health in children.

## Figures and Tables

**Table 1:** Laboratory and anthropometric parameters of our population of children with acquired hypothyroidism on thyroid hormone replacement therapy.

	*No. of Subjects*	*Mean (Max–Min)*
**TSH (microUI/mL)**	212	3.26 (19.66–0)
**fT3 (ng/L)**	197	3.97 (12–0.15)
**fT4 (ng/dL)**	207	5.24 (18.7–5.51)
**Glucose (mg/dL)**	125	79.94 (123–37)
**Triglycerides (mg/dL)**	104	87.65 (490–33)
**Total cholesterol (mg/dL)**	109	160.47 (293–95)
**HDL cholesterol (mg/dL)**	96	51.51 (103–28)
**LDL cholesterol (mg/dL)**	97	91.75 (168–40)
**Age (years)**	212	9.78 (21–1)
**Weight *z*-score**	212	0.3 (3.43–−6.34)
**BMI *z*-score**	212	0.53 (3–−3.8)
**Height *z*-score**	212	−0.28 (3.12–−4.93)

**Table 2:** Laboratory and anthropometric parameters of children with acquired hypothyroidism on thyroid hormone replacement therapy and children with normal thyroid function (control group).

*Patients with Hypothyroidism*		*Control Group*	*p Value*
**Glucose (mg/dL)**	82.9	78.4	0.045
**Triglycerides (mg/dL)**	106.7	79.9	0.046
**Age (years)**	12.3	7.1	<0.001
**Mean weight *z*-score**	0.15	0.46	0.135
**Mean BMI *z*-score**	0.49	0.58	0.615
**Mean height *z*-score**	−0.53	−0.02	0.01

## Data Availability

No new data were created or analyzed in this study; data sharing is not applicable to this article.
